# The carbon footprint of external beam radiotherapy and its impact in health technology assessment

**DOI:** 10.1016/j.ctro.2024.100834

**Published:** 2024-07-31

**Authors:** Chloé Dupraz, Coline Ducrot, Benoit Allignet, Gregory Delpon, Anthony Alexis, Ariane Lapierre, Stéphane Supiot, David Ali, Max Piffoux

**Affiliations:** a Oncologie médicale, Hospices civils de Lyon, France; b Département de Chirurgie Orthopédique, Hôpital Femme Mère Enfant, Hospices Civils de Lyon, Bron, France; c Département de radiothérapie, Centre Léon Bérard, Lyon, France; d Univ Lyon, INSA‐Lyon, Université Claude Bernard Lyon 1, Laboratoire CREATIS UMR 5220, U1294 Lyon, France; e Département de Physique Médicale, Institut de Cancérologie de l’Ouest, site Saint-Herblain, France; f Laboratoire SUBATECH, UMR 6457 CNRS-IN2P3, IMT Atlantique, Nantes, France; g Service de radiothérapie, Hôpital lyon sud, Pierre bénite, France; h Département de Radiothérapie, Institut de Cancérologie de l’Ouest, site Saint-Herblain, France; i Laboratoire US2B, CNRS UMR 6286, Université de Nantes, Nantes, France; j Centre de Radiothérapie de Versailles, France; k Direction de la Recherche Clinique et de l’Innovation, Centre Léon Bérard, Lyon, France

**Keywords:** Carbon footprint, Hypofractionation, Health technology assessment

## Abstract

**Background:**

The major drivers of carbon dioxide (CO_2_eq) emissions of external beam radiation therapy (EBRT) are not well known and limit our ability to initiate mitigation strategies.

**Material and methods:**

We describe the carbon footprint of four typical centers. We explore direct EBRT associated factors such as the impact of fractionation and use of MRI-LINAC, as well as indirect factors (e.g. patient rides). Treatment strategy related CO_2_eq emissions are included in a health technology assessment analysis that takes into account CO_2_eq emissions.

**Results:**

A typical EBRT treatment emits from 185 kgCO_2_eq to 2066 kgCO_2_eq. CO_2_eq emissions are mostly driven by (i) accelerator acquisition and maintenance (37.8 %), (ii) patients and workers rides (32.7 %), (iii) drugs and medical devices (7.3 %), (iv) direct energy consumption (6.1 %), and (v) building and bunker construction (5.6 %) with a substantial heterogeneity among centers. Hypofractionation has a strong impact to mitigate emissions. MRI-LINAC is associated with a substantial increase in CO_2_eq emissions per fraction and requires ultra hypofractionation in 5 fractions to achieve a similar carbon footprint compared to 20 fractions treatment schemes. The expected limited small increase in toxicities due to hypofractionation (when existing) are in the same range as avoided detrimental effects to future people’s health thanks to CO_2_eq mitigation.

**Conclusion:**

Carbon footprint of EBRT is not neglectable and could be mitigated. When safely feasible, hypofractionation is one of the main factors to decrease this impact. Taking into account CO_2_eq emissions has a substantial impact on the health technology assessment of EBRT, favoring hypofractionated regimens.

## Highlights

• A typical radiotherapy treatment emits from 185 to 2066 kgCO2eq in France.
• CO2eq emissions are mostly driven by accelerator acquisition and maintenance as well as patients and workers rides.
• Hypofractionation has a strong impact on mitigating carbon footprint.
• Hypofractionation related toxicities are usually lower than damages avoided to future people thanks to CO2eq mitigation.

## Introduction

Global warming and climate change will have a strong impact on the healthcare sector in the future. The impact of the latter on global warming and greenhouse gas (GHG) emissions is not anecdotal as it accounts for about 9 % of US GHG emissions [1]. Most countries pledged to drastically reduce their carbon emissions in the future, including healthcare. In a particular sector, better understanding the main drivers of carbon emissions allows us to focus on the most efficient actions to mitigate them and to take this into account when comparing treatment strategies.

How decisions made by healthcare professionals can impact the carbon footprint of healthcare are not easy to conceive. The French healthcare system emits 46 MtCO_2_eq, representing about 8 % of national emissions [2], i.e. about ∼203 tCO_2_eq/year per physician (226.000 in France) or 0.68tCO_2_eq/person in France. In the US, it represents about 590 tCO_2_eq/physician/year [1]. The annual mean carbon emission of a French citizen is estimated to 9.9 tCOeq [3] whereas the estimated worldwide annual emission required to control global warming to 1.5 °C is 2tCO_2_eq/person [4].

Cheung et al [5] reported the impact of the COVID pandemic on emissions in their radiotherapy (RT) center focusing on the carbon footprint related to patient’s travel, linear accelerator power usage and personal protection equipment consumption, showing a 39 % decrease in carbon emissions to reach a mean emission of about 273 kgCO_2_eq/patient. Unfortunately, restricting the focus and not taking into account all other CO_2_eq emission sources may have led to a significant underestimation of the real carbon footprint of radiotherapy. Similarly, Lichter et al recently published a life cycle analysis of radiotherapy in the US that does not take into account accelerator maintenance and purchase, nor data storage [6]. Chuter et al. also proposed an estimation in the UK that was restricted to patient travel, imaging, electricity, SF6 emissions and personal protection equipment [7]. Other authors compared the interest of EBRT to surgery in lung cancer from a carbon footprint perspective, although based on conservative estimates for radiotherapy [8]. We therefore used the carbon footprint methodology to estimate the complete carbon footprint of radiotherapy treatments.

We investigated the carbon impact of external beam radiotherapy (EBRT) in four different representative centers, compared MRI LINAC to conventional photon-based RT and explored the relation between these carbon emissions and their effect on future people health.

## Materials and methods

### Estimation of carbon footprints

Radiotherapy is delivered in a certain number of fractions, usually ranging from 1 to 40. The carbon footprint of RT was defined by a fixed part that does not vary according to the treatment strategy, and a variable part corresponding to a RT fraction, which is multiplied by the overall number of fractions. Carbon footprint was calculated in four different facilities representing different kind of radiotherapy centers: the public university hospital Lyon Sud, the public peripheral hospital of Bourg-en-Bresse, the Institut de cancérologie de l’Ouest and the private peripheral clinic of Versailles, all treating >1,000 patients/year.

The carbon footprint of RT encompassed energy consumption (electricity, gas, building, etc), purchases (including machines, drugs, medical devices, etc), trips (patients and workers ride, work trips, etc), immobilizations (building, bunkers, IT, material), waste, laundry, imaging and biology. Each post was quantified in the fixed and variable part. As an example, all expenses related to planning computer tomography (CT) or treatment calculation, was attributed to the fixed part whereas the use of accelerators was attributed to the variable part.

When possible, *direct* conversion was used (carbon footprint of an object multiplied by the number used). When not available, emission factors (kgCO_2_eq/€) were used to allow the conversion between a cost (€) and a carbon footprint (kgCO_2_eq). The emission factors were obtained directly from the literature [9] or calculated from literature data when not available. Data were obtained over a 1-year period in order to limit season-related fluctuations.

### RT accelerators, scanners and maintenance

To the best of our knowledge, no detailed analysis of the carbon footprint of accelerators for EBRT encompassing accelerators maintenance and construction is available among analysis proposed in the literature [5], [6], [7].

We estimated the carbon footprint impact of this subpart by calculating a specific emission factor (kgCO_2_eq/€). We found two emission factors from Varian in 2019 [10] and Elekta in 2021 [11] that both contained the *production*, *use phase* and *recycling* of accelerators. We recalculated emission factors for accelerators by subtracting the *use phase* that is taken into account more precisely in our analysis. The recalculated mean emission factor (conversion using November 2022 rates) without use phase is 0.245kgCO_2_eq/€ (±0.083). These companies are dedicated to RT equipment, their sales are mainly related to linear accelerators, scanners or maintenance services. This estimate is therefore relatively unbiased by other kinds of revenues. Of note, since RT facilities usually buy accelerators as well as maintenance contracts from these companies, the emission factor reflects both these services. This factor is in good agreement with the proposed emission factor of 0.39 kgCO_2_eq/€ for machinery, installation and repair proposed by the French national environment agency ADEME [9].

### Patients and worker’s transport

Distance and mode of transport of patients and workers were collected and transformed in carbon footprint using emission factors per kilometer for each transport method, i.e. 0.401kgCO_2_eq/km in ambulance, 0.216kgCO_2_eq/km in personal car/taxi, 0.258 kgCO_2_eq/km for short distance plane, 0.05kgCO_2_eq/km for public transports (ADEME[9] and Shift Project[2]).

### Accounting of material consumption

Material consumption spendings were multiplied by the associated ADEME emission factor (in kgCO_2_/€) in order to obtain the carbon footprint. These spendings also encompassed medical consumption at home (i.e. prescribed drugs and medical devices) related to the RT treatment that were estimated from representative patient prescriptions and expert opinion.

We used the following emission factors: 0.315 kgCO_2_eq/€ for medical devices, 0.5 kgCO_2_eq/€ for drugs, 0.01 kgCO_2_eq/€ for medical biology (mean of emission factors from various exams [12]), 0.6kgCO_2_eq/€ for services and manufactured products, 0.4kgCO_2_eq/€ for electronic products, 0.4kgCO_2_eq/€ with an expected time of use of 5 years for IT and servers products. When feasible, other spendings were accounted by *direct conversion*: 0.79 kgCO_2_eq per patient [13] for histology, 9.2 kgCO_2_eq/exam per CT-scan, 17.5 kgCO_2_eq/exam [14] per MRI and the same value for PET scanner (in the absence of data in the literature), 0.6kgCO_2_eq/kg for laundry, 0.353 kgCO_2_eq/kg for household waste and 0.955 kgCO_2_eq/kg for high-risk waste. Fiducial insertion under echography accounted for 33kgCO_2_ [15] and material needed accounted for as for medical devices.

### Direct energy consumption

Electricity and gas were the only energies used in our hospitals. The direct consumption was quantified using annual consumption. If not available, the consumption per meter square in the whole hospital was used for gas consumption (heating). Emission factors for electricity (0.0569kgCO_2_eq/kWh) and gas (0.227kgCO_2_eq/kWh) are from ADEME.

### Building and bunker

Carbon emission from building construction in the health system from ADEME [2] were used. Bunkers construction was quantified using the estimated amount of cement used to build a bunker multiplied by the emission factor of cement (398kgCO_2_eq/m^3^ of reinforced concrete, amortized in 60 years).

### Hospitalization

The estimation of the total carbon footprint of a day hospital was estimated using the same methodology in the medical oncology department of CHU Lyon Sud (42.29 kgCO_2_eq/day, personal unpublished data).

### Confidence intervals

Intervals containing 95 % of the distribution, representing the incertitude surrounding the estimation were estimated using bootstrap with 10,000 iterations with R (CRAN). When a mean value for all centers is calculated, intercenter variation is added. Normal distribution was used for all parameters except rides, with a common standard deviation based on both the incertitude of carbon footprint estimation (ranging from 10-50 % based on ADEME proposed incertitude and the intercenter variation when applicable.

### Carbon emission induced damage to healthcare in the future due to global warming

**Fig. 1:**
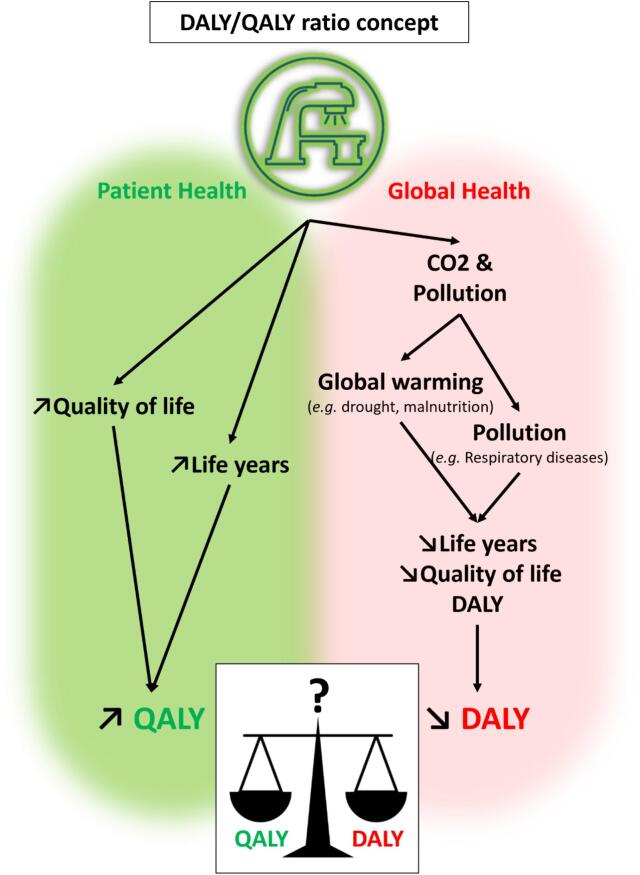
The DALY/QALY ratio concept. A treatment strategy may have both an impact on patient health (in QALY) and an impact on GHG emissions that translates into future health damages (in disability adjusted life years, DALY).

Disease adjusted life years (DALYs) are a time-based measure that combines years of life lost due to premature mortality and years of healthy life lost due to time lived in states of less than full health as a consequence of a particular threat to health [16]. DALY are quite similar to QALY in many ways (see Feng et al. for more details [17]) although QALY usually represent gains in quality-of-life adjusted life years compared to DALY that usually represent losses for a population. Global warming may be considered as a threat to actual and future health. We used already published conversion factors from the ReCiPe model to estimate the future health damage associated to the amount of CO_2_eq emitted due to a treatment [18], [19]. This model estimates that incremental emission of 80 CO_2_eq metric tons (tCO_2_eq) induces one DALY, i.e. leads to the loss of one year of life in good health in the future. Of note, this estimation of the ReCiPe model comes from a scenario stated as “egalitarian” that considers that DALYs lost today or in 500 years have the same value. These models rely on climatic models to predict global warming per region and its impact on health. As an example, global warming induces higher risks of drought in many regions, leading to lower agricultural yield and malnutrition. Said otherwise, a treatment that leads to incremental emissions of 8 tCO_2_eq is expected to induce the loss of 0.1 DALY in the future with this model. In order to compare QALY gains for patients due to the treatment and DALY losses in the future induced by the treatment emissions, we used the DALY/QALY ratio (see Fig. 1).

## Results

### Carbon footprint of external beam radiotherapy

**Fig. 2:**
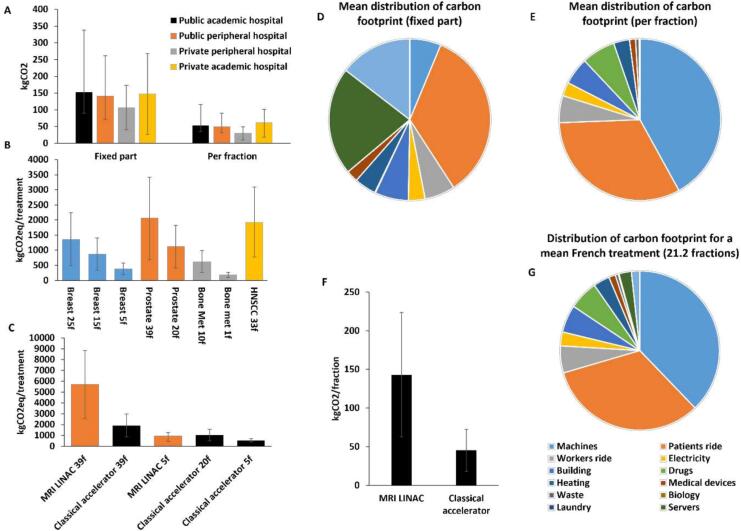
Carbon footprint of RT treatments. (A) Carbon footprint of each center divided into fixed and per fraction parts. (B) Carbon footprint of various treatment schemes for common cancers. (C) Carbon footprint related to the treatment of localized prostate cancer depending on the accelerator used. (D) Mean distribution of the carbon footprint of the radiotherapy fixed part. (E) Mean distribution of the carbon footprint per fraction. (F) Carbon footprint per fraction depending on the type of accelerator. (G) Mean distribution of the carbon footprint for a typical treatment (21.2 fractions).

The mean *fixed* part (treatment planning, medical physics, tumor biopsy, initial imaging, pre-treatment consultations, etc, Fig. 2A) is estimated to 137.5 kgCO_2_eq (±41.5) and the mean *variable* part is estimated to 48.9 kgCO_2_eq/fraction (±17.9). Fixed carbon footprint is mainly related to patients' travels, data storage and servers as well as imaging (Fig. 2D). Carbon footprint per fraction represents the most important part of the overall footprint, and is mostly related to patients’ rides as well as acquisition and maintenance of accelerators (Fig. 2E, G). Interestingly, the private peripheral center had an overall lower carbon footprint (respectively 106 kgCO_2_eq in fixed part and 29.8 kgCO_2_eq/fraction) than the three other hospitals with similar *fixed* and *variable* emissions (respectively ±141–153kgCO_2_eq and ±50–62kgCO_2_eq/fraction).

The private hospital's lower carbon footprint is largely explained by a strong decrease of patient rides footprint in proportion of the total carbon footprint. This center is located in a large urban area and attracts patients from a much lower distance compared to the two other centers (mean distance 21.8 km versus 60–80 km for the others). Furthermore, the private center also had a lower emission in the workers' ride, electricity consumption, building and heating subparts, explained by a smaller building surface, as well as the use of electricity to heat the facility.

More generally, mean RT carbon footprint for a typical French treatment (21.2 fractions [20]) is about 1174 ± 379 kgCO_2_, and is mostly related to accelerators purchase and maintenance (37.8 %), workers and patients' rides (38.2 %), drugs and medical devices (7.3 %), electricity and heating (6.1 %) and buildings construction (5.6 %).

A typical radiation oncologist is expected to treat about 211 patients per year (207,000 patients per 979 radiation oncologists in France [21]. It represents a mean of 247 ± 80 tCO_2_eq per radiation oncologist per year. A typical center treating 1000 patients is expected to emit 1174 ± 379 tCO_2_eq/year.

### Impact of hypofractionation

Based on the estimation of carbon footprint of the fixed and variable part of RT, we calculated the carbon footprint of complete RT treatment strategies depending on the fractionation used (Fig. 2C). Reducing the number of fractions reduces almost linearly the carbon footprint of a treatment strategy. Breast cancer treatment in 25 fractions, 15 fractions and 5 fractions emits 1,358 kgCO_2_eq (95 %CI 486–2,247), 868 kgCO_2_eq (95 %CI 343–1,407) and 378 kgCO_2_eq (95 %CI 189–575), respectively. Prostate cancer treatment in 39 fractions or 20 fractions emits 2,066 kgCO_2_eq (95 %CI 685–3,425) versus 1,135 kgCO_2_eq (95 %CI 415–1,826). Bone metastasis treatment in 10 fractions emits 626 kgCO_2_eq (95 %CI 270–989), whereas it only emits 186 kgCO_2_eq (95 %CI 95–273) when treated in a single fraction.

### Application case on MRI LINAC

Due to daily adaptive treatment in MRI LINAC, this machine is expected to deliver radiation more accurately to limit side effects to surrounding tissues. Although the fixed part is relatively similar when using conventional or MRI linear accelerator, variable fraction is largely impacted by the carbon footprint of the accelerator, leading to the emission of 142 kgCO_2_eq/fraction (95 %CI 63–224) for MRI-LINAC versus 46 kgCO_2_eq/fraction (95 %CI 18–72) for classical accelerators. When keeping the same amount of fraction as in a common accelerator, *e.g*. 39 in prostate cancer, it leads to a major increase in amount of CO_2_eq per treatment from 1,911 kgCO_2_eq (95 %CI 846–2,962) to 5,706 kgCO_2_eq (95 %CI 2,581–8,844, Fig. 2F). On the other hand, in particular situations, MRI LINAC allows highly hypo-fractionated strategies [22], [23]. In these hypo-fractionated strategies, MRI LINAC with 5 fractions competes with 20 fractions on classical accelerators in terms of carbon footprint (932 kgCO_2_eq/treatment, 95 %CI 444–1,262) versus 1,046 kgCO_2_eq/treatment (95 %CI 494–1,587) for conventional accelerators. It is anyway more CO_2_eq-emitting than 5-fractions schemes on classical accelerators (365 kgCO_2_eq/treatment, 95 %CI 205–520). We explore how this estimation may be extrapolated to other countries in supplementary data 1.

## Health technology assessment of hypofractionation strategies taking into account CO_2_eq emissions

### Localized prostate cancer in France

We used data from K. Zhou et al in the French setting [24] that investigated the cost effectiveness of a hypo-fractionated treatment (HT, 20 fractions) versus a normo-fractionated treatment (NT, 39 fractions) for localized prostate cancer in France. QALY were higher in the HT arm at + 0.044 QALY (although non-significant). Using HT versus NT regimen decreases CO_2_eq emissions by 864 kgCO_2_eq using the previously described emission model.

Using the ReCiPe 2016 model[18] to translate emissions in future damage to health, 864 kgCO_2_eq translates into −0.011 disability adjusted life years (DALY, a metric similar to QALY) lost in the future due to climate change. The 20-fraction regimen has a negative incremental DALY/QALY ratio (−25 %) compared to the 39-fraction regimen, meaning that it is both better for patients and for future health (Fig. 1 and Table 1).

**Table 1:** Medical and medico-ecologic analysis of hypofractionation strategies versus normal fractionation strategies in different settings.

Treatment strategy	Incremental QALY	Incremental Cost	Incremental kgCO_2_eq	Incremental DALY	Incremental DALY/QALY
Localized prostate cancer [24], French setting					
20 fractions versus 39 fractions	0.044	−1,296€	−864	−0.011	**−25 %**
Localized Prostate cancer [25], US setting					
39 fractions versus 5 fractions	0.011	+$9,900	+1,371	0.017	**+155 %**
Localized breast cancer [26], UK setting					
Partial breast cancer in 5 fractions versus whole breast in 15 fractions	0.017	−1,750£	−489	−0.006	**−35 %**
Whole breast cancer in 5 fractions versus whole breast in 15 fractions	0.05	−2,162£	−489	−0.006	**−12 %**

### Localized prostate cancer in the USA

Similarly, Parikh et al [25] evaluated the cost effectiveness of normo-fractionated treatment (NT, 39 fractions), hypo-fractionated (HT, 20 fractions) and ultra-hypo-fractionated (UHT, 5 fractions, taking into account the need for fiducials) for the treatment of localized prostate cancer in the US setting. NT led to the highest QALY gains in their model (4.09 QALY) versus 4.04 QALY for HT and 4.08 QALY for UHT (Table 1). Compared to NT, HT and UHT led to reduction of 864 and 1371 kgCO_2_eq emission, respectively translating in 0.011 and 0.017 DALY.

When taking CO_2_eq emissions into account using our model, the incremental QALYs gained by NT versus UHT (+0.011 QALY) are relatively smaller than the estimated DALYs induced by incremental CO_2_eq emissions (0.017 DALY). The DALY/QALY ratio is equal to 155 %, meaning that GHG emissions may induce more harm for future generations than gains for patients, or at least be in the same order of magnitude (Table 1 and Fig. 1).

### Localized breast cancer in the UK

Glynn et al [26] evaluated the interest of either partial or whole breast cancer irradiation in 5 or 15 fractions in patients eligible for partial breast cancer irradiation in the UK setting. In their analysis, 5-fraction partial breast RT is both less costly and associated with the best quality of life (Table 1). In their analysis, using 5 fractions instead of 15 also allows to decrease carbon emissions by 489kgCO_2_eq (i.e. saving 0.006 DALY). Therefore, compared to the base case whole breast irradiation in 15 fractions, partial breast treatment in 5 fractions is associated with a negative DALY/QALY ratio, meaning in that case that it is both better for patients and for future people. Similarly, in patients non eligible to partial breast cancer, whole breast irradiation in 5 fractions versus 15 fractions was already considered dominant (both saving £2,162 and allowing to gain 0.05 QALYs), but it also allows to save 489kgCO_2_eq (i.e. saves 0.006 DALY).

## Discussion

We report the first estimation, to the best of our knowledge, of the external beam RT treatment strategies complete carbon footprint. The treatment induced carbon emission is mostly driven by (i) accelerator acquisition and maintenance (37.8 %), (ii) patients and workers rides (32.7 %), (iii) drugs and medical devices (7.3 %), (iv) direct energy consumption (6.1 %) and (v) building and bunker construction (5.6 %). There is a substantial heterogeneity among centers, mainly driven by patient rides distance, but also by choices regarding accelerator type and energy mix. Compared to the restricted estimations from Cheung et al [5] that focused on patients travel, linear accelerator power usage and personal protection equipment consumption that led to an estimation of about 270 kgCO_2_eq/treatment, our results show a higher complete carbon footprint of 1,174 kgCO_2_ per treatment. This may be partly explained by the fact we take into account accelerator use but also their construction and maintenance in our analysis. It has a major impact as spending 1 million € to buy an accelerator has a carbon footprint of 245 tCO2eq (as estimated by accelerator companies). Once distributed on each patient, it adds an important contribution on the accelerator purchase and maintenance part. Lichter et al recently published a life cycle analysis of radiotherapy in the US that does not take into account accelerator maintenance and purchase, nor data storage that are major contributors in our analysis. They find a mean emission of 4.3 tCO_2_eq for a 25-fraction treatment [6], yet still larger than ours as USA is a more carbon intensive environment [27], [28]. Lastly, Chuter et al obtained an estimation of 75–226 kgO_2_eq while restricting their analysis to patient travel, imaging, electricity, SF6 emissions and personal protection equipment [7]. Although diminishing the accelerator manufacturer's carbon footprint cannot be directly controlled by radiation oncologists, investment in new and costly accelerators like MRI-LINAC has a limited efficiency from a carbon footprint and medico-economic perspective apart if used exclusively to perform highly hypo-fractionated strategies that may not be feasible with classical accelerators. We did not evaluate the carbon footprint of carbon ions and proton-based radiotherapy but one may guess similar findings may be obtained.

A large body of literature discusses the relative risks of hypo-fractionated regimens compared to normo-fractionated ones that largely exceed this article scope. This complex problem was up to date mostly discussed taking into account the benefit and risks for the patient in its particular context, its quality of life and time that may be impacted by daily rides to the hospital. GHG accounting has up to date not been accounted for in these analyses although it may have substantial impact.

Hypo-fractionation, even in worst case scenarios where hypofractionation is associated with more toxicities for patients still leads to similar effects in terms of overall QALY gains [24], [25], [26]. Hypo-fractionation allows to mitigate CO_2_eq emissions leading to a decrease in carbon emissions, climate change impact and subsequent induced future detrimental health effects on the population (DALYs). Overall, hypofractionation seems to be rather less costly, leads to a small gain in QALY, rendering it cost effective [24], [25], [26] from a medico economic point of view. When taking into account its mitigating effect on carbon emissions on top of it, it is even more of interest from a public health and societal point of view.

Our multicentric study's major limits are (i) that it is limited to the French setting at the moment but explained how our estimation may be extrapolated to other countries and what are the key factors to take into account, (ii) is limited to external beam X-ray based radiotherapy, (iii) is based on a hybrid direct and costing method, (iv) we make the hypothesis that emissions per fraction will be similar in terms of imaging per fraction in normo- or hypo-fractionated schemes, whereas hypofractionation usually require higher monitor units, and are often more image guided and (v) that a substantial uncertainty surrounds the value of carbon emission estimated per treatment strategy (about 30 %, encompassing inter-patient, inter-center and emission factors uncertainty).

## Conclusion

A usual RT treatment has an important carbon footprint of ∼200-2,000kgCO_2_eq in France. This can be largely reduced and there are important differences between centers. (Ultra)-hypo-fractionated strategies significantly decrease the carbon emissions. The limited increase in toxicities (if any) due to hypofractionation are in the same range of magnitude as avoided detrimental effects induced by climate change to future people health thanks to CO_2_eq mitigation.

## Author contributions

All authors gathered data from their centers, M.P and D.A performed the first analysis, all authors then contributed to a critical review of the analysis, M.P wrote the first draft of the manuscript, all authors reviewed and accepted the final manuscript.

## Data availability statement

All data apart the ones regarding accelerators contracts (under confidential agreement) are available upon request to the corresponding author.

## Declaration of competing interest

The authors declare that they have no known competing financial interests or personal relationships that could have appeared to influence the work reported in this paper.
