# Supplementary material for: The carbon footprint of external beam radiotherapy and its impact in health technology assessment
Source: Clin Transl Radiat Oncol. 2024 Jul 31;48:100834. doi: 10.1016/j.ctro.2024.100834 (PMC11359761; doi:10.1016/j.ctro.2024.100834)
Supplement: Supplementary Data 1 [file mmc1.docx]

**Supplementary data**

**Extrapolation to other countries and settings**

Extrapolation to other non-French settings cannot be done directly. We focused on the most important subparts of the carbon footprint (patients and workers' ride, accelerator construction and maintenance, electricity, heat consumption as well as building construction) to explore how extrapolation to other countries may be performed. Patients and workers' rides may vary a lot from one region to another both in distance and in type of transport used and may be adapted using adequate national emission factors. Energy used to run the facility has a major impact on the RT carbon footprint. In France, an electricity kWh has an estimated relatively low carbon footprint of 56.9 gCO_2_eq/kWh thanks to a high fraction of nuclear energy whereas it is estimated to be about 348 gCO_2_eq/kWh in Germany in 2021 [1] and 433 gCO_2_eq/kWh in the US in 2019 [2], leading respectively to an increase of 17% and 22% kgCO_2_eq per patient if using the same electricity consumption. Emission factors for accelerator installation and maintenance is estimated based on manufacturers' data that sell their products worldwide and may therefore probably be applied to any setting. Building construction is expected to be relatively similar from one country to another and nationally adapted emission factors are usually available.

**Supplementary references**

[1] European Environment Agency. Greenhouse gas emission intensity of electricity generation. Https://WwwEeaEuropaEu/Data-and-Maps/Daviz/Co2-Emission-Intensity-13/#tab-Chart_4 2023.

[2] U.S. Environmental Protection Agency. AVERT, U.S. national weighted average CO2 marginal emission rate, year 2019 data. Washington, DC: 2020.
